# Characterization of the Oligomerization and Aggregation of Human Serum Amyloid A

**DOI:** 10.1371/journal.pone.0064974

**Published:** 2013-06-04

**Authors:** Sanket Patke, Saipraveen Srinivasan, Ronak Maheshwari, Sunit K. Srivastava, J. Javier Aguilera, Wilfredo Colón, Ravi S. Kane

**Affiliations:** 1 Department of Chemical and Biological Engineering, Rensselaer Polytechnic Institute, Troy, New York, United States of America; 2 Center for Biotechnology and Interdisciplinary Studies, Rensselaer Polytechnic Institute, Troy, New York, United States of America; 3 Department of Chemistry and Chemical Biology, Rensselaer Polytechnic Institute, Troy, New York, United States of America; Universidad de Granada, Spain

## Abstract

The fibrillation of Serum Amyloid A (SAA) – a major acute phase protein – is believed to play a role in the disease Amyloid A (AA) Amyloidosis. To better understand the amyloid formation pathway of SAA, we characterized the oligomerization, misfolding, and aggregation of a disease-associated isoform of human SAA – human SAA1.1 (hSAA1.1) – using techniques ranging from circular dichroism spectroscopy to atomic force microscopy, fluorescence spectroscopy, immunoblot studies, solubility measurements, and seeding experiments. We found that hSAA1.1 formed alpha helix-rich, marginally stable oligomers *in vitro* on refolding and cross-beta-rich aggregates following incubation at 37°C. Strikingly, while hSAA1.1 was not highly amyloidogenic *in vitro*, the addition of a single N-terminal methionine residue significantly enhanced the fibrillation propensity of hSAA1.1 and modulated its fibrillation pathway. A deeper understanding of the oligomerization and fibrillation pathway of hSAA1.1 may help elucidate its pathological role.

## Introduction

Reactive amyloidosis or Amyloid A (AA) amyloidosis is a condition in which amyloid deposits of the protein serum amyloid A (SAA) appear in organs like the spleen, liver, and kidney [1]–[4]. Reactive amyloidosis generally accompanies other conditions that induce chronic inflammation such as rheumatoid arthritis and atherosclerosis [1], [5], [6]. Serum amyloid A is an acute-phase reactant found circulating in the blood plasma primarily associated with high density lipoproteins (HDL) [7], [8]. SAA has been shown to play critical roles in a wide range of functions including cholesterol transport, HDL metabolism, and host defense [9]–[12]. A persistently high level of SAA in the plasma and specific tissues, arising as a result of inflammation, is believed to be the prerequisite for the development of reactive amyloidosis [2],[4],[5].

SAA is found in almost all vertebrates investigated and exhibits a high degree of similarity between species [13], [14]. The human genome includes four SAA genes, of which SAA1 and SAA2 are acute-phase proteins, SAA3 is a pseudogene and SAA4 is a constitutively expressed protein [14], [15]. Characterization of amyloid A (AA) protein in tissues of patients suffering from reactive amyloidosis has revealed the predominant deposition of SAA1 and its proteolytic fragments as amyloid deposits [1], [16]. In particular, a specific allele of SAA1, human SAA1.1 (hSAA1.1) (previously called SAA1α) was shown to be a principal component of AA amyloid deposits [16], [17]. Pioneering work by Parmelee et al. [18] led to the amino acid sequence analysis of hSAA1.1 isolated from the high density lipoprotein fraction of a pool of human sera with elevated levels of Amyloid A immunoreactivity. In addition to its role in reactive amyloidosis, hSAA1.1 has also been shown to play a role in other pathological conditions including but not limited to asthma [19], chronic obstructive pulmonary disease [20], and thrombosis [21].

Despite the significant amount of work done to understand the physiological role of SAA [12], [22]–[25], the exact role of SAA misfolding and aggregation in reactive amyloidosis remains unclear. As a first step towards addressing these issues, we characterized the oligomerization and fibrillation properties of hSAA1.1 *in vitro*. We note that we have used delipidated (apo) hSAA1.1 for our studies. While physiologically, SAA is predominantly associated with HDL [8], [26], it has been shown that SAA can exist *in vivo* in lipid-free form [5], [27]. Moreover, since our goal was to probe the intrinsic structure and fibrillation propensities of hSAA1.1, the absence of HDL was necessary for these *in vitro* studies.

Interestingly, most previous studies discussing the pathological importance of hSAA1.1 use a protein expressed in *E. coli* [28] or obtained commercially [19], [20], [29], [30], that carries a N-terminal methionine, a residue which is absent in the SAA protein found in amyloid deposits *in vivo* [7], [16], [18]. We therefore used both hSAA1.1 without the N-terminal methionine (hSAA1.1) and with the N-terminal methionine (referred to as MetSAA1.1). Such a side-by-side comparison of the commonly studied version of the protein (MetSAA1.1) and the physiologically relevant isoform (hSAA1.1) would help provide a more comprehensive understanding of the oligomerization and fibrillation properties of hSAA1.1.

Based on size exclusion chromatography and circular dichroism spectroscopy, we found that both MetSAA1.1 and hSAA1.1 form alpha-helix-rich oligomers immediately upon refolding. While incubation of both the proteins at 37°C resulted in the formation of cross-beta-rich aggregates with similar aggregation kinetics, there were some striking differences between the fibrillation propensities and pathways of both the proteins, suggesting a critical role for the amino-terminal methionine. Aggregation of MetSAA1.1 resulted in the formation of soluble and spherical oligomers in the early stages and long and insoluble “rod-like” amyloid fibrils in the later stages. In contrast, hSAA1.1, even upon prolonged incubation at 37°C, existed as soluble spherical oligomers and short curvilinear aggregates, all of these aggregates being much smaller and having a different conformation relative to the aggregates formed by MetSAA1.1. Interestingly, the amyloid fibrils formed by MetSAA1.1 were able to seed the fibrillation of MetSAA1.1 while hSAA1.1 did not exhibit seeding properties further suggesting the importance of the methionine residue in the aggregation of the protein. This insight into the intrinsic oligomerization and fibrillation propensities of the AA Amyloidosis-relevant isoform of human SAA will provide a useful starting point in understanding the pathological activity of this protein.

## Materials and Methods

### MetSAA1.1 and hSAA1.1 Expression and Purification

MetSAA1.1 was purified following a procedure described previously for murine SAA [31]. Briefly, MetSAA1.1 cDNA was cloned into a pET21-a(+) vector between the NdeI and BamHI sites and transformed into *E. coli* BL21 (DE3) pLysS-competent cells. The cells were grown, expressed, and lysed using standard procedures. Cation exchange buffer (8 M urea/20 mM sodium acetate, pH 4.7) was used as lysis buffer and the lysate was purified using SP Sepharose cation exchange column (GE Healthcare Biosciences) on an AKTA purifier UPC 10 FPLC (GE Healthcare Biosciences) using an elution buffer (8 M urea/20 mM sodium acetate/400 mM NaCl, pH 4.7) to isolate the protein. The relevant fractions, based on analysis by sodium dodecyl sulfate – polyacrylamide gel electrophoresis (SDS-PAGE), were pooled and desalted by dialyzing them against anion exchange buffer (8 M urea/20 mM Tris, pH 8.3). This desalted fraction was then loaded on a DE52 anion exchange column and eluted with an anion exchange elution buffer (8 M urea/20 mM Tris/400 mM NaCl, pH 8.3) on an AKTA FPLC (GE Healthcare Biosciences) using a 10−65% salt gradient. The relevant fractions, based on analysis by SDS-PAGE, were pooled and concentrated via several rounds of ultrafiltration and then loaded on a HiLoad 16/60 Superdex 200 preparative grade column (GE Healthcare Biosciences) preequilibrated with size exclusion chromatography (SEC) buffer (8 M urea/20 mM Tris/200 mM NaCl, pH 8.3), and the relevant fractions were collected and concentrated using an Amicon ultrafiltration cell.

We used a protocol similar to one used in one of our previous studies to purify hSAA1.1 [32]. Similar to MetSAA1.1, hSAA1.1 cDNA was also cloned into a pET21-a(+) vector between the NdeI and BamHI sites. We first expressed the protein with a N-terminal his-tag and a recognition site for tobacco etch virus (TEV) ((His)_6_–TEV-SAA) in *E.coli* BL21 (DE3) pLysS-competent cells and lysed the cells in lysis buffer (8 M urea/20 mM Tris, pH 8.3). The ultracentrifuged and filtered lystate was then loaded on a Histrap column (GE Healthcare Biosciences) and (His)_6_–TEV-SAA was eluted using immobilized metal affinity chromatography (IMAC) elution buffer (8 M urea/20 mM Tris/400 mM imidazole, pH 8.3). The relevant fractions containing (His)_6_–TEV-SAA were then pooled and loaded on a SEC column (HiLoad 16/60 Superdex 200, GE Healthcare Biosciences) pre-equilibrated with SEC buffer (8 M urea/20 mM Tris/200 mM NaCl) to isolate pure (His)_6_-TEV-SAA. Purified (His)_6_–TEV-SAA was then extensively dialyzed against Tris buffer (20 mM Tris/1 mM EDTA, pH 7.4) to remove urea from the protein solution. TEV protease was then used to cleave dialyzed (His)_6_–TEV-SAA (1∶20 TEV:SAA) resulting in the generation of cleaved protein (hSAA1.1) and uncleaved (His)_6_–TEV-SAA. Protein sequence of (His)_6_–TEV-SAA indicating TEV cleavage site has been included in the supporting information section. Figure S1 in File S1 summarizes the TEV proteolysis reaction monitored as a function of time. TEV cleaves (His)_6_-TEV-hSAA1.1 at a site between “Q” and “R” residues yielding hSAA1.1, sequence of which begins with “R” and with no residues from TEV remaining in the protein sequence. TEV proteolysis was quenched by adding a solution containing urea and NaCl to bring up the final concentration of the solution to 5 M urea and 500 mM NaCl. The protein solution containing hSAA1.1, uncleaved (His)_6_–TEV-SAA, and (His)_6_–TEV was then dialyzed against a buffer containing 8 M urea and 20 mM Tris, pH 8.3 to remove traces of EDTA. The resulting protein solution in IMAC buffer (8 M urea/20 mM Tris, pH 8.3) was then passed through a Histrap column (GE Healthcare Biosciences) and hSAA1.1 was collected in the eluant. The unbound protein was collected, concentrated, and further purified using a gel filtration column (HiLoad 16/60 Superdex 200, GE Healthcare Biosciences) preequilibrated with SEC buffer (8 M urea/20 mM Tris/200 Mm NaCl) to obtain hSAA1.1. Relevant fractions obtained after SEC were pooled and concentrated using Amicon ultrafiltration cells. The purity of MetSAA1.1 and hSAA1.1 was confirmed by SDS-PAGE and electrospray ionization−mass spectroscopy (ESI-MS). Protein concentration was determined by measuring the absorbance at 280 nm and using calculated values of the extinction coefficients.

### Refolding of hSAA1.1

Both hSAA1.1 and MetSAA1.1 proteins were refolded by dialyzing purified protein in SEC buffer (8 M urea/20 mM Tris-HCl, 200 mM NaCl, pH 8.3) against Tris buffer (20 mM Tris-HCl, pH 8.3) using a 3 kDa MWCO membrane at 4°C. Dialysis was performed overnight with multiple buffer changes during the course of the experiment. Buffer used for dialysis was pre-cooled to 4°C prior to dialysis and the apparatus was maintained at 4°C at all times.

### Size Exclusion Chromatography (SEC)

All SEC studies were performed at 4°C on a Superdex 200 10/300 GL analytical grade column (GE Healthcare Biosciences) using an AKTA purifier UPC 10 (GE Healthcare Biosciences). Molecular weight (MW) standards were used to calibrate the SEC column. Approximately 30 µL of refolded proteins (20 µM) obtained after dialysis against Tris buffer, was loaded in the analytical SEC column pre-equilibrated with TBS (20 mM Tris/200 mM NaCl, pH 8.3, 4°C). Elutions were performed at a flow rate of 0.5 mL/min. All the experiments involving characterization of the oligomeric state of refolded hSAA1.1 were performed at 4°C.

### Circular Dichroism (CD) Spectroscopy

Far-UV CD experiments were performed using a J-815 CD spectrometer (Jasco). Approximately 250 µL of SAA solution, diluted to a final concentration of 20 µM using Tris buffer (20 mM Tris-HCl, pH 8.3, 4°C), was used for the measurement of CD spectra and also for the thermal denaturation experiments. The diluted protein samples were loaded in a rectangular quartz cuvette (1 mm pathlength) and 10 spectra were collected from 200 nm to 260 nm and later averaged. Other experimental settings were as follows: Data pitch, 0.5 nm; Scanning mode, Continuous; Scanning speed, 50 nm/min; Bandwidth, 1 nm. Thermal denaturation experiments on hSAA1.1 were performed by measuring the molar ellipticity of hSAA1.1 with molar ellipticity at 222 nm as the reference. The experimental settings for thermal denaturation experiments were as follows: Temperature range, 4–80°C; Data pitch, 2°C; Slope, 1°C/min.

### Tryptophan Fluorescence Assay

Tryptophan fluorescence measurements were performed using a Spex FluoroLog Tau3 fluorometer (Horiba). Approximately 3 mL of SAA solution, diluted to a final concentration of 10 µg/mL using Tris buffer (20 mM Tris-HCl, pH 8.3, 4°C), was used for the measurement of fluorescence spectra and also for the tryptophan fluorescence-based thermal denaturation experiments. The diluted protein samples were loaded in a rectangular quartz cuvette (10 mm pathlength) and 10 spectra were collected from 320 nm to 400 nm and later averaged. Other experimental settings were as follows: Data pitch, 0.5 nm; Scanning mode, Continuous; Scanning speed, 50 nm/min; Bandwidth, 1 nm; Emission slit width, 5 nm; Excitation slit width; 3 nm. Thermal denaturation experiments on both proteins were performed by measuring the fluorescence intensity and emission maxima of the proteins with emission maxima of freshly refolded proteins used as the reference. The experimental settings for thermal denaturation experiments were as follows: Temperature range, 4–80°C; Data pitch, 2°C; Slope, 1°C/min.

### Thioflavin T (ThT) Fluorescence Assay

For ThT fluorescence assay, a solution containing freshly refolded protein (20 µM) in Tris buffer (20 mM Tris-HCl, pH 8.3, 4°C) was incubated at 37°C without agitation in an Eppendorf Mastercycler (Eppendorf). At desired time intervals, 5 µL of this solution were mixed with 5 µL ThT dye solution (260 µM) and 90 µL of glycine-NaOH buffer (50 mM glycine, pH 8.5) and measurements were made on a Safire II microplate reader (Tecan). Analysis was done in triplicates. The experimental settings used were as follows: excitation wavelength, 440 nm; emission wavelength, 485 nm; excitation bandwidth, 5 nm; emission bandwidth, 5 nm; Number of scans, 6.

### Congo Red Binding Assay

We followed the procedure described in Srinivasan et al. [33] with some minor modifications. A Congo red stock solution (10 mM) was prepared by dissolving Congo red in Tris:ethanol (9∶1) at pH 8.3. Ethanol was used to prevent micelle formation. The stock solution was filtered three times using a 0.2 µm filter. From this stock solution, a diluted solution of Congo red (20 µM) was prepared. Accurate concentrations of the diluted Congo red solution was determined by absorbance spectroscopy at 505 nm and using the Beer-Lambert plot (ε = 5.53×10^4 ^cm^−1 ^M^−1^). Two hundred microliters of the diluted Congo red solution (20 µM) were then mixed with 100 µL of a protein solution (that had been previously incubated for 24 h at 37°C at a starting concentration of 20 µM) and the resulting mixture was incubated for 30 min at RT. Congo red binding was confirmed by measuring a UV absorbance spectrum from 400–600 nm using a Lambda 35 UV spectrophotometer (Perkin – Elmer), with subtraction of the baseline spectrum of the Tris buffer. The final spectrum was compared to that of a solution containing Congo red (13.3 µM) but no protein.

### Measurement of Soluble Protein Concentration

For assays involving the measurement of soluble protein in the protein sample, solutions containing freshly refolded protein (20 µM) in Tris buffer (20 mM Tris-HCl, pH 8.3, 4°C) were incubated at 37°C without agitation in an Eppendorf Mastercycler (Eppendorf). At desired time intervals, 50 µL of sample was centrifuged at 12,000 rpm (13,523×g) at 37°C for 10 min to separate the soluble fraction (supernatant) from the insoluble fraction (pellet). Soluble protein concentration was determined by measuring the absorbance of the supernatant at 280 nm and using the calculated value of the extinction coefficient.

### Atomic Force Microscopy (AFM)

AFM measurements were performed as described previously [31]. Briefly, SAA samples at different incubation times were diluted 10–20 fold in Tris buffer (20 mM Tris-HCl, pH 8.3) and then applied on freshly cleaved mica. The samples were incubated on the mica for 15 min and the mica was then washed 3–4 times with filtered water. AFM was performed at room temperature using an Asylum Research MFP 3D AFM instrument (Asylum Research), operating in tapping mode in air. Specifications of the silicon cantilevers (AC240TS, Olympus) used for AFM analysis were as follows: spring constant, 1.8 N/m; tip radius, <10 nm; resonant frequency, 70 kHz; tip height, 14 µm; cantilever thickness, 2.8 µm.

### Immunoblot Assay

Immunoblot assay using A11 and OC antibodies were performed as described previously [34]. Solution containing freshly refolded protein (20 µM) in Tris buffer (20 mM Tris-HCl, pH 8.3, 4°C) was incubated at 37°C without agitation in an Eppendorf Mastercycler (Eppendorf). 3–4 µL of this sample was spotted on a nitrocellulose membrane (GE Healthcare Biosciences). The membrane was allowed to dry overnight at room temperature (RT). The membrane was then blocked with 10% nonfat dry milk (in PBS) for 2 h, followed by a wash with PBST for 10 min. The blots were then incubated with A11 (Invitrogen) or OC (Millipore) antibodies (dilutions for the antibodies were: 1∶700 for A11 and 1∶1000 for OC in 5% nonfat dry milk in PBS) for 1 h at RT, followed by a wash with PBST for 10 min. The membrane was then incubated with anti-rabbit horseradish peroxidase-conjugated secondary antibody for 1 h at RT, followed by a wash with PBST for 10 min. After washing, the blots were exposed to ECL Western blotting substrate (Thermofisher) and then developed. Isotype rabbit antibodies were used for control immunoblot experiments.

## Results

### MetSAA1.1 and hSAA1.1 Form Alpha Helical Oligomers on Refolding

**Figure 1:**
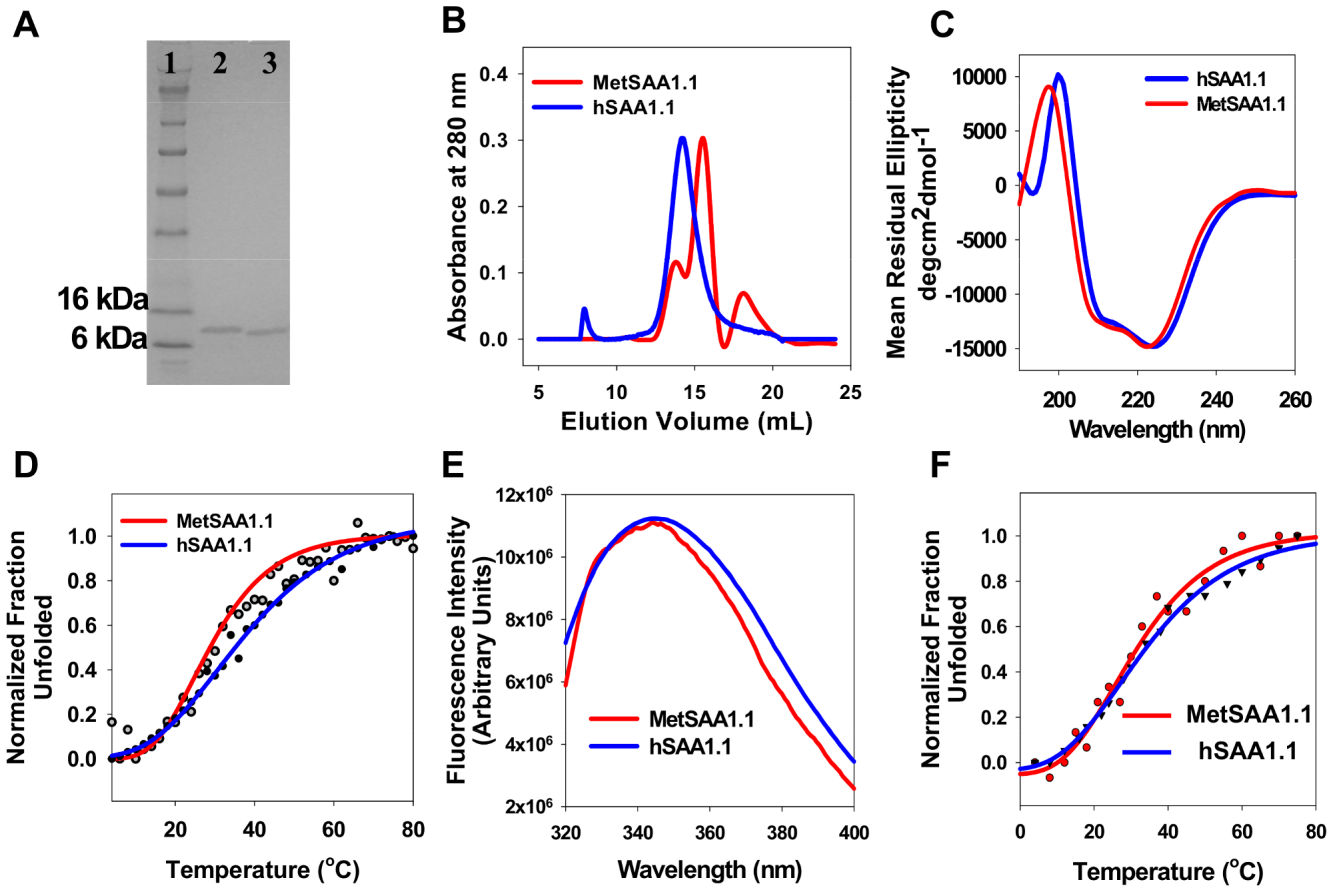
Characterization of hSAA1.1 and MetSAA1.1 by SDS-PAGE, SEC, far UV-CD, tryptophan fluorescence, and thermal denaturation studies. (A) SDS-PAGE gel (lanes: 1, protein ladder; 2, hSAA1.1; 3, MetSAA1.1 (B) SEC elution profiles of MetSAA1.1 (red solid line) and hSAA1.1 (blue solid line); (C) far UV-CD spectra of MetSAA1.1 (red solid line) and hSAA1.1 (blue solid line); (D) Thermal denaturation profiles of MetSAA1.1 (red solid line) and hSAA1.1 (blue solid line) (E) Tryptophan emission spectra of MetSAA1.1 (red solid line) and hSAA1.1 (blue solid line); (F) Tryptophan fluorescence-based thermal denaturation profiles of MetSAA1.1 (red solid line) and hSAA1.1 (blue solid line). The concentration of protein used in all the experiments was 20 µM. All experiments were performed at 4°C.

Both versions of SAA were expressed, purified and refolded using the protocols described in the experimental procedures section (See Supporting Information for more details about the sequence, UniProtKD ID, estimated MW, and extinction coefficient values of the proteins). In order to have a consistent starting point for all the studies, the experiments were performed on freshly refolded protein. By doing so, we ensured that conformational changes associated with the storage of proteins for extended periods of time did not affect the consistency of the results [35]. Given the marginal stability of hSAA1.1, as discussed later in the text, refolding was performed at 4°C. Refolded protein was assessed for its purity by using SDS-PAGE (Fig. 1A) and ESI-MS (data not shown). As expected, MetSAA1.1 (MW ∼ 11.81 kDa) and hSAA1.1 (MW ∼ 11.68 kDa) showed a single band with MW between 6 and 16 kDa (Fig. 1A). Amino-terminal MS-MS analysis of the first 7 amino acids yielded MRSFFSF for MetSAA1.1 and RSFFSFL for hSAA1.1. These amino acid sequences are also consistent with the sequences of the protein available in the literature [8], [12], [18], [28].

We characterized the quaternary structure of MetSAA1.1 and hSAA1.1 using size exclusion chromatography (SEC). The SEC chromatogram for MetSAA1.1 showed a prominent peak with an elution volume of ca. 15.6 mL and two smaller peaks corresponding to elution volumes of ca. 13.9 mL and ca. 18.1 mL (Fig. 1B). Based on calibration performed using MW standards and on our previous work with murine SAA where a detailed characterization of the oligomeric conformation was performed [31], [32], [35], the elution volume of 15.6 mL is consistent with a tetramer while elution volumes of 13.9 mL and 18.1 mL are consistent with octamers and monomers, respectively. In contrast, the hSAA1.1 elution profile predominantly exhibited a single peak the location of which varied between 13.8–14.5 mL corresponding to a mixture of octamers and hexamer (Fig. 1B). This ability to form both octamers and hexamers is similar to that for murine SAA2.2, as we have reported previously [35]. We refer to these hSAA1.1 and MetSAA1.1 oligomers as *in vitro* “native-like” oligomers because they were obtained immediately after refolding the denatured protein. These conformations might not necessarily represent the oligomeric conformation of lipid-free or HDL-associated native SAA in vivo. Additionally, assignment of precise molecular size to the oligomers, based on SEC elution volumes, was made with the principal assumption that the eluting species were globular in shape.

We then probed the oligomeric conformations of both the proteins refolded at different concentrations to study the effect of protein concentration on its oligomerization properties. Specifically, both the proteins were refolded at 75 µM, 20 µM, and 8.5 µM concentrations and their quaternary structure was analyzed by SEC at each of these concentrations. As shown in Figure S2A in File S1, while tetramers (primary oligomers) and octamers were the preferred oligomeric conformation of MetSAA1.1 at 75 and 20 µM concentrations, refolding the protein at 8.5 µM concentrations resulted in the formation of a considerable population of MetSAA1.1 monomers in addition to tetramers and octamers. Similarly, hSAA1.1 also predominantly formed a mixture of octamers and hexamers (primary oligomers) at 75 and 20 µM concentrations. However, minor quantities of tetramers and a considerable population of hSAA1.1 monomers were observed in addition to hSAA1.1 octamers at a concentration of 8.5 µM (Fig. S2B in File S1). Collectively, these results point to a strong dependence of protein oligomeric conformation on protein concentration. While both the proteins formed very similar oligomers at 75 and 20 µM protein concentrations, they exhibited a high propensity to exist as their respective monomers at 8.5 µM concentration. These results might suggest that oligomerization of both the proteins is favorable at high protein concentrations.

Next, we used far-UV circular dichroism (CD) spectroscopy to characterize the secondary structure of these “native-like” SAA oligomers. As seen in Figure 1C, oligomers of both proteins were primarily α-helical, as indicated by the two negative peaks centered at 222 and 208 nm – hallmarks of a protein with high alpha-helical content. We also characterized the thermal stability of both proteins (20 µM) using CD-based thermal denaturation studies. As seen in Figure 1D, the data can be fitted to a sigmoidal curve. The T_m_ value for MetSAA1.1 was ca. 29°C. Low values of melting temperatures suggest marginal stability of these “native-like” MetSAA1.1 oligomers. Similar calculations for hSAA1.1 indicated that the melting temperature of this protein was ca. 35°C. Thus, addition of a hydrophobic methionine residue at the N-terminus of hSAA1.1 resulted in a slight decrease in the thermal stability of the protein. We note that the values of the melting temperature (T_m_) obtained from these experiments are only for comparison purposes, as the thermal unfolding of the proteins was found to be irreversible (data not shown). We also observed a dependence of protein refolding concentration on the thermal stabilities of both the proteins. While MetSAA1.1 refolded at 8.5 µM concentration yielded a T_m_ value of 25°C (Fig. S1C in File S1), hSAA1.1 refolded at the same concentration yielded a T_m_ value of 27°C (Fig. S1D in File S1). These T_m_ values mark a slight reduction in the thermal stabilities for both the proteins (from 29°C to 25°C for MetSAA1.1 and from 35°C to 27°C for hSAA1.1) as a result of refolding at lower concentrations.

Next we compared the tertiary structure of both hSAA1.1 and MetSAA1.1. Since both the proteins have three tryptophan residues each, comparison of tertiary structures was done by comparing the intrinsic fluorescence values of both the proteins by following tryptophan fluorescence. Protein solutions refolded at 20 µM concentrations were diluted in Tris buffer (20 mM Tris, pH 8.3) to a final concentration of 10 µg/mL and fluorescence measurements were made at 4°C as described in the materials and methods section. As shown in figure 1E, “native-like” oligomers of both the proteins exhibit similar tryptophan emission spectra with similar quantum yields and wavelength maxima (ca. 345 nm). Similarities in the emission spectra for both the proteins suggest that they might have similar tertiary structures. We also monitored the changes in the tertiary structure of both the proteins by following tryptophan emission upon temperature increase. We observed a red shift in emission maxima upon temperature increase with the peak shifting from ca. 345 nm to ca. 356 nm for both the proteins (data not shown). As shown in figure 1F, the data for normalized change in the emission maxima with 345 nm (emission maxima of “native-like” oligomers) as a reference can be fitted to a sigmoidal curve. The T_m_ values for MetSAA1.1 and hSAA1.1 calculated by monitoring changes in the tryptophan emission maxima upon temperature increase was ca. 31°C and ca. 33°C respectively. To probe the influence of the concentration of the proteins on their tertiary structures, we measured tryptophan emission spectra for proteins refolded at concentrations of 20 µM and 8.5 µM. As shown in Figure S2E in File S1, the emission spectrum of MetSAA1.1 “native-like” oligomers refolded at 20 µM yielded an emission maximum at 345 nm, while the spectrum for MetSAA1.1 “native-like” oligomers refolded at 8.5 µM had emission maximum at 351 nm. This red shift in the emission maximum suggests a greater exposure of tryptophan residues for the protein refolded at lower concentration. Similar results were obtained for hSAA1.1, with an emission maximum at 345 nm for protein refolded at 20 µM and 351 nm for protein refolded at 8.5 µM (Fig. S2F in File S1).

We then performed structural studies to investigate whether the starting SAA proteins in 8 M urea prior to refolding against Tris buffer possessed any residual structure. Specifically, we monitored the secondary and quaternary structure of both the proteins in 8 M urea using far-UV CD and size exclusion chromatography techniques respectively. Quaternary structure analysis on SAA samples containing 8 M urea using SEC (column equilibrated in 8 M urea/20 mM Tris/200 mM NaCl) suggested that both the proteins eluted as a single peak with an elution volume of ca. 19.1 mL representing a monomeric species (Fig. S3A and B in File S1). Additionally, we performed far-UV CD studies on protein solutions in 8 M urea but were unable to obtain a reliable spectrum due to heavy interference of high concentration urea solutions with CD spectrometer. We therefore performed CD measurements at lower urea concentrations which could be expected to yield low urea interference and high reliability results. As shown in Figure S3C and S3D in File S1, both the proteins upon refolding formed alpha-helix rich oligomers (0 M urea). Most of the secondary structure was lost in 2 M urea as indicated by the small absolute mean residual ellipticity values around 220 nm, suggesting marginal chemical stability of the “native-like” oligomers formed by both the proteins. We have previously reported that murine SAA2.2 loses its alpha-helical characteristics in ∼ 2 M urea [36]. Collectively, these results suggest that 8 M urea is a condition harsh enough for the protein to retain any structural features of “native-like” oligomers and therefore the starting protein prior to refolding against Tris buffer is expected to not possess any residual structure.

Additionally, we performed “cross-purification” studies to ascertain that the minor differences in the conformations and thermal stabilities of the “native-like” oligomers formed by both the proteins was a result of the inherent properties of both the proteins and not an artifact of differences in the manipulation histories involved in the purification of these proteins. Specifically, following purification of MetSAA1.1 (referred to as “regular” MetSAA1.1) using a sequential cation exchange chromatography, anion exchange chromatography, and size exclusion chromatography procedure, the purified protein in size exclusion chromatography buffer (8 M urea/20 mM Tris/200 mM NaCl, pH 8.3) was passed through a Histrap Ni-NTA column (please refer to materials and methods sections for more details about the column) and MetSAA1.1 proteins samples (referred to as “cross-purified” MetSAA1.1) were collected in the flowthrough. Similarly, following purification of hSAA1.1 (referred to as “regular” hSAA1.1) using the procedure described in the materials and methods section (IMAC, SEC, TEV proteolysis, IMAC, SEC in that sequence), the purified protein was further purified by using sequential cation exchange chromatography and anion exchange chromatography procedures to obtain “cross-purified” hSAA1.1. Relevant buffer exchanges and ultrafiltration steps were followed prior to each of the ion exchange chromatography steps. Both the “cross-purified” proteins were then refolded by using the same refolding protocol which has been described in the materials and methods section. “Cross-purification” therefore ensured that both the proteins, which we expressed using the same expression vector, same bacteria, and refolded using identical dialysis procedures, were also exposed to the same ions and resins during their purification and therefore had similar manipulation histories right from protein expression until protein refolding. We found that both “cross-purified” MetSAA1.1 and “regular” MetSAA1.1, upon refolding at 20 µM concentrations, formed a heterogeneous population consisting of tetramers (primary oligomer), octamers/hexamers, and monomers (Figure S4 in File S1). Similarly, as shown in Figure S4B in File S1, even “cross-purified” hSAA1.1 refolded at 20 µM concentrations predominantly formed octamers/hexamers upon refolding – an oligomeric conformation similar to the one formed by refolded “regular” hSAA1.1 (Fig. S5 in File S1). Taken together, these results suggest that the minor differences in the oligomeric conformation formed by both the proteins were not due to the differences in their manipulation histories.

### Both MetSAA1.1 and hSAA1.1 Form Cross-Beta-rich Aggregates with Contrasting Solubilities

Next, we investigated the ability of MetSAA1.1 and hSAA1.1 “native-like” oligomers to aggregate at 37°C. We use the term “aggregation” to refer to higher-order self-assembly of SAA beyond the “native-like” oligomers. We chose to study SAA aggregation at 37°C since our previous studies using mouse isoforms of SAA yielded amyloid fibrils at this temperature [31], [32], [37], [38]. We used the ThT fluorescence assay to investigate the aggregation kinetics of hSAA1.1 and MetSAA1.1. Thioflavin T dye shows a strong binding affinity to cross- beta structures and exhibits enhanced fluorescence upon binding to such structures [39].

**Figure 2:**
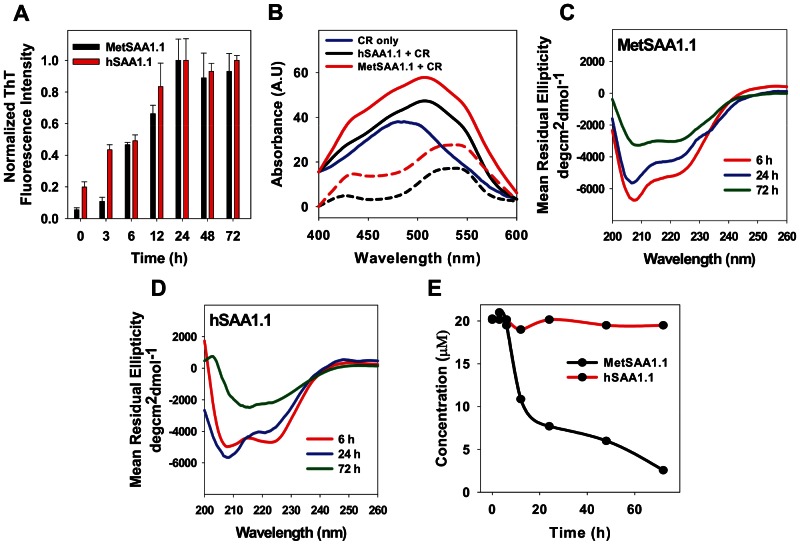
Characterization of aggregation of MetSAA1.1 and hSAA1.1 by the ThT Fluorescence assay, Congo red binding assay, far UV CD, and solubility assay. (A) ThT fluorescence intensity profile for MetSAA1.1 (black bars) and hSAA1.1 (red bars); (B) Congo red absorbance spectra for Congo red only (blue solid line); Congo red plus MetSAA1.1 sample (red solid line); MetSAA1.1 difference spectra (red dash line); Congo red plus hSAA1.1 sample (black solid line); hSAA1.1 difference spectra (black dash line); (C) Far UV CD spectra of MetSAA1.1 samples incubated at 37°C for 6 h (red solid line), 24 h (blue solid line), and 72 h (green solid line); (D) Far UV CD spectra of hSAA1.1 samples incubated at 37°C for 6 h (red solid line), 24 h (blue solid line), and 72 h (green solid line); (E) solubility profile for MetSAA1.1 (black solid line) and hSAA1.1 (red solid line). The starting concentration of protein was 20 µM. All assays were performed after the proteins were allowed to aggregate at 37°C.

We induced aggregation of the “native-like” protein oligomers by incubating the proteins (starting concentration 20 µM) at 37°C and monitored the intensity of ThT fluorescence as a function of time. As seen in Figure 2A, MetSAA1.1 aggregation was characterized by a time-dependent increase in the ThT fluorescence intensity suggesting a transformation of the “native-like” oligomers into aggregates rich in cross-beta structure. A measurable “lag time” of ca. 3 h was observed, and the ThT fluorescence intensity reached its maximum value by ca. 24 h, after which there was no significant change in the fluorescence intensity. Next, we performed similar ThT fluorescence experiments using hSAA1.1. hSAA1.1 showed similar ThT binding kinetics, with a time-dependent increase in ThT fluorescence intensity followed by saturation of ThT signal by ca. 24 h (Fig. 2A). Similar ThT fluorescence intensity profiles for both proteins suggested the formation of cross-beta-rich aggregates with similar aggregation kinetics.

To ascertain that the aggregates formed by both the proteins have cross-beta structures, we used the well-established Congo red binding assay [33], [40]. Amyloid aggregates with cross-beta structure exhibit a characteristic red shift in their absorption spectrum upon binding to Congo red [33]. We induced aggregation by incubating the proteins at 37°C. Since both the proteins had reached a ThT fluorescence maximum by ca. 24 h, we chose to test the ability of the aggregates formed after 24 h to bind to Congo red. While free Congo red solution exhibited absorption spectra with absorption maxima at ca. 485 nm, aggregates of both MetSAA1.1 and hSAA1.1 formed after 24 h exhibited absorption maxima at ca. 506 nm upon Congo red binding (Fig. 2B). The absorption shift is even more evident from the difference spectrum (bound Congo red minus free Congo red), which exhibits an absorption maxima at ca. 540 nm. These results are consistent with the presence of cross-beta structures in the aggregates formed by both MetSAA1.1 and hSAA1.1.

Next, we monitored changes in the secondary structures of MetSAA1.1 and hSAA1.1 at different time intervals following incubation at 37°C. While the “native-like” oligomers formed by MetSAA1.1 were rich in alpha-helical content (Fig. 1C), the CD spectra obtained after incubation of the protein at 37°C for 6 h and 24 h exhibited a shoulder at 222 nm and a deep minimum around 207 nm (Fig. 2C red and blue lines). These CD spectra were consistent with those from a previous study reporting the conversion of predominantly alpha-helix-rich structures of two isoforms of murine SAA to a mixture of alpha-helix, random coil, beta-sheet, and beta-turn structures [41]. Interestingly, the spectrum for MetSAA1.1 after 72 h displayed enhanced alpha-helicity relative to samples obtained at earlier time points (Fig. 2C green line). However, caution must be exerted in interpreting this data as the protein sample was found to contain high levels of insoluble aggregates at this time (see discussion below). Therefore the CD spectrum obtained for MetSAA1.1 after 72 h may represent primarily the soluble protein fraction and cannot be used to draw inferences about the secondary structure of the “entire” protein population. As shown in Figure 2D, far UV CD studies on hSAA1.1 samples obtained after 6 h and 24 h yielded results similar to those observed for MetSAA1.1. Aggregates of hSAA1.1 obtained after these incubation times were also characteristic of a mixture containing alpha-helical, random coil, beta-sheet, and beta-turn structures. However, in the case of this protein, 72 h samples, which did not contain any insoluble precipitates, exhibited CD spectra with a minimum centered at 215 nm, providing direct evidence of beta-sheet structures.

These studies were followed by experiments to check the solubilities of the aggregates formed during this 72 h period. Samples from both the proteins obtained after different incubation times were spun down at 12,000 rpm at 37°C for 10 min to separate the soluble protein fraction (supernatant) from the insoluble fraction (pellet) [42]. Concentrations of the soluble protein fractions were determined by measuring the absorbance at 280 nm using known values of the extinction co-efficients of both proteins. As shown in Figure 2E, MetSAA1.1 aggregation was characterized by a time-dependent decrease in the concentration of protein present in the supernatant, suggesting a conversion of soluble oligomers into insoluble protein aggregates. In contrast, hSAA1.1 aggregates were soluble even after ca. 72 h (Fig. 2E). These results suggest that there are some fundamental differences between the solubilities of the aggregates formed by MetSAA1.1 and hSAA1.1.

Collectively, these results suggest that the aggregation of MetSAA1.1 involved the conversion of the alpha helix - rich “native-like” oligomers to soluble oligomers in the early stages of aggregation and eventually into insoluble cross-beta-rich aggregates. On the other hand, aggregation of hSAA1.1 was characterized by the conversion of the “native-like” oligomers into soluble oligomers rich in cross-beta structure. These critical differences point to some fundamental differences between the aggregation pathways of the two proteins, although they differ by only a single methionine residue at the N-terminus.

### Characterization of Aggregation by AFM and Immunoblots

**Figure 3:**
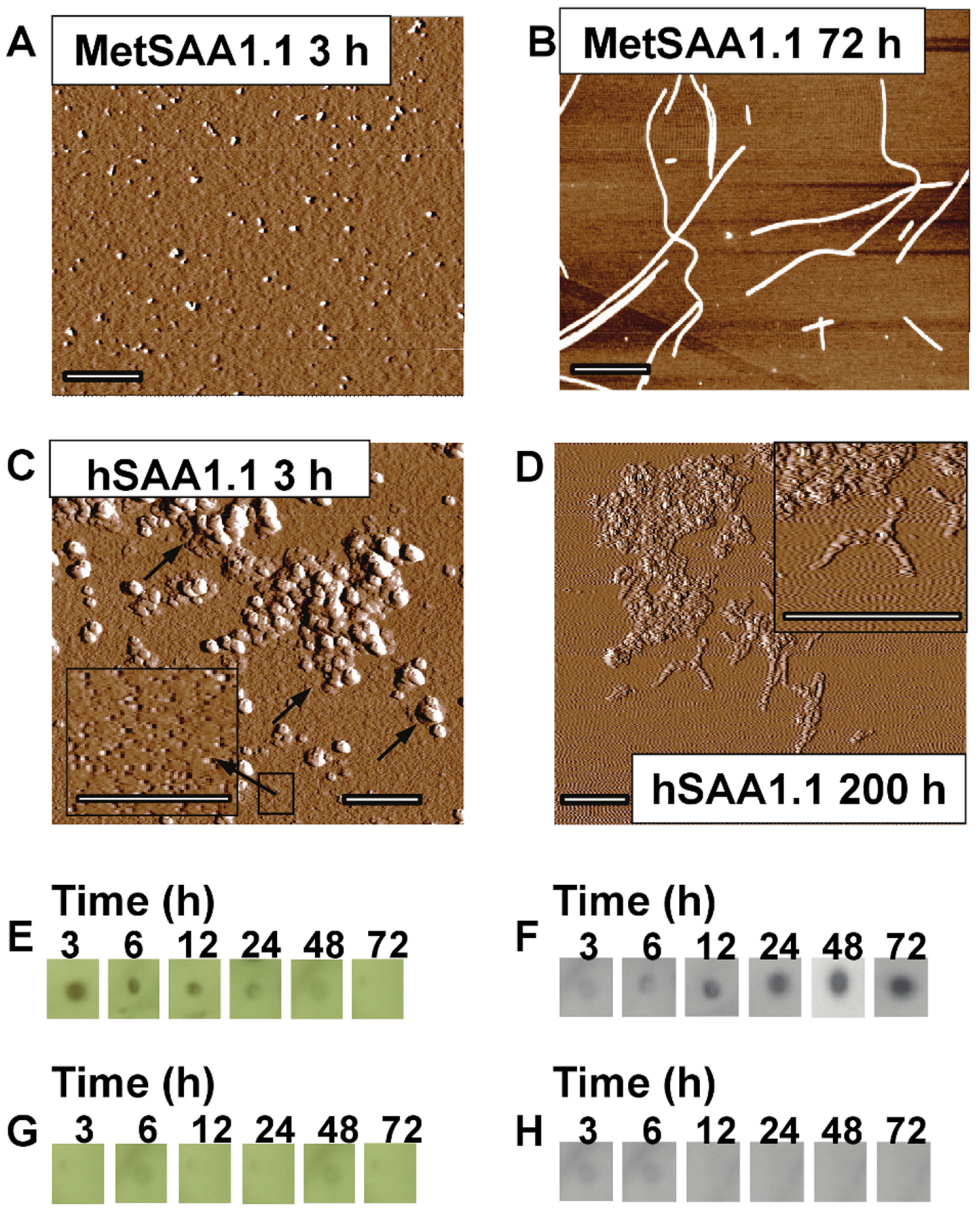
Biophysical characterization of aggregates formed by MetSAA1.1 and hSAA1.1. AFM analysis of (A) MetSAA1.1, 3 h, 37°C; (B) MetSAA1.1, 72 h, 37°C; (C) hSAA1.1, 3 h, 37°C; (D) hSAA1.1, 200 h, 37°C; Immunoblot analysis of aggregates formed by MetSAA1.1 using (E) A11 antibody and (F) OC antibody; Immunoblot analysis of aggregates formed by hSAA1.1 using (G) A11 antibody and (H) OC antibody. All scale bars for AFM images represent 1 µm.

Intrigued by the observation that both versions of hSAA1.1 exhibited similar aggregation kinetics, but differed in the solubilities of their aggregates, we decided to further characterize the species formed during aggregation. We performed AFM studies for morphological characterization of the protein aggregates. We incubated the MetSAA1.1 “native’-like” oligomers (see AFM in Fig. S5A in File S1) at 37°C and performed AFM on protein samples obtained after different time intervals. As shown in Figure 3A, “spherical” aggregates with heights in the range of ∼ 4–6 nm were predominantly observed following the incubation of MetSAA1.1 at 37°C for 3 h. Further incubation up to 6 h at 37°C resulted in the formation of short fibrils with “rod-like” architecture (Fig. S6A in File S1and inset) with heights ranging from 4–6 nm and lengths ranging from 10–15 nm. Small “spherical” prefibrillar aggregates, similar to the ones observed after 3 h, were observed along with these short fibrils. Incubating MetSAA1.1 at 37°C for 24 h resulted in the formation of large “rod-like” fibrils (Fig. S6B in File S1). By 72 h, MetSAA1.1 formed 15–20 µm long full-length mature amyloid fibrils (Fig. 3B). Although the heights of most of these fibrils were ∼ 4–6 nm, taller fibrils with heights of ∼ 10–12 nm were also observed. This observation would be consistent with the length-wise stacking of full-length fibrils over each other. Collectively, the AFM studies and solubility assay results for MetSAA1.1 suggest that the spherical aggregates formed by this protein might represent the population of soluble oligomers while the “rod-like” fibrillar structures might represent the population of insoluble aggregates.

We then performed atomic force microscopy studies on aggregates formed by hSAA1.1. We incubated the hSAA1.1 “native’-like” oligomers (see AFM in Fig. 5SB) at 37°C and performed AFM on protein samples obtained after different time intervals. Aggregation of hSAA1.1 at 37°C resulted in the formation of spherical aggregates after ca. 3 h (Fig. 3C) that had a very strong propensity to cluster and form large assemblies of spherical aggregates (Fig. 3C inset). The height of individual spherical aggregates was ca. 1 nm and they were much smaller than the spherical aggregates formed by MetSAA1.1 after ca. 3 h. Similar clusters of spherical aggregates with similar dimensions were observed after ca. 12 and 48 h following incubation (Supporting information Fig. S6C–D in File S1). However, small curvilinear fibril-like aggregates could be seen emerging from these clusters in the samples obtained after ca. 200 h. Interestingly, unlike MetSAA1.1 which formed full-length amyloid fibrils, hSAA1.1 failed to form full-length amyloid fibrils even after 200 h incubation (Fig. 3D). Collectively, the AFM studies and solubility assay results for hSAA1.1 suggest that the spherical aggregates and small curvilinear fibril-like structures formed by this protein may be soluble in the buffer.

We next tested whether MetSAA1.1 and hSAA1.1 aggregates shared any structural similarity with the soluble oligomers and amyloid fibrils formed by some commonly studied amyloid proteins. Glabe and co-workers have identified antibodies that recognize the soluble oligomers [43] and fibrils [44] formed by most common amyloid proteins. Since aggregation of both MetSAA1.1 and hSAA1.1 also involved the formation of soluble oligomers (Figs. 2B, 3A, and 3C) or insoluble fibrils (Figs. 2B and 3B), we tested whether these aggregates had any epitopes for oligomer-specific (A11) and fibril-specific (OC) antibodies using dot blot assays. MetSAA1.1 “native-like” oligomers did not exhibit any binding to A11 antibody (data not shown). However, as shown in Figure 3E, oligomer-specific A11 antibody showed moderate binding to all the MetSAA1.1 samples from ca. 3 h until ca. 12 h and no binding thereafter. In contrast, the fibril-specific OC antibody showed very weak binding until ca. 12 h, and strong binding for all samples thereafter (Fig. 3F). These results indicate that the aggregates formed by MetSAA1.1 share conformational similarities with their counterparts formed by most commonly studied amyloid proteins [43], [44]. Strikingly, neither A11 (Fig. 3G) nor OC antibodies (Fig. 3H) were able to bind significantly to the aggregates formed by hSAA1.1. Additionally, there was no A11 binding observed for the “native-like” oligomers formed by hSAA1.1 (data not shown). While absence of OC binding is consistent with the AFM results suggesting the inability of hSAA1.1 to form full-length amyloid fibrils, the absence of A11 binding to the soluble and spherical aggregates formed by hSAA1.1 might suggest that they do not share structural similarities with the soluble oligomers formed by most amyloid proteins and also to their counterparts formed by MetSAA1.1. Alternatively, it is possible that “clustering” of hSAA1.1 oligomers to form larger assemblies adversely affects the accessibility of the A11 antibody to epitopes on individual oligomers, thus resulting in decreased A11 antibody binding. Nevertheless, these results obtained from AFM and immunoblot studies further confirm that the N-terminal methionine has a critical influence on the fibrillation pathway of the protein.

### Seeding Properties of MetSAA1.1 and hSAA1.1 Aggregates

**Figure 4:**
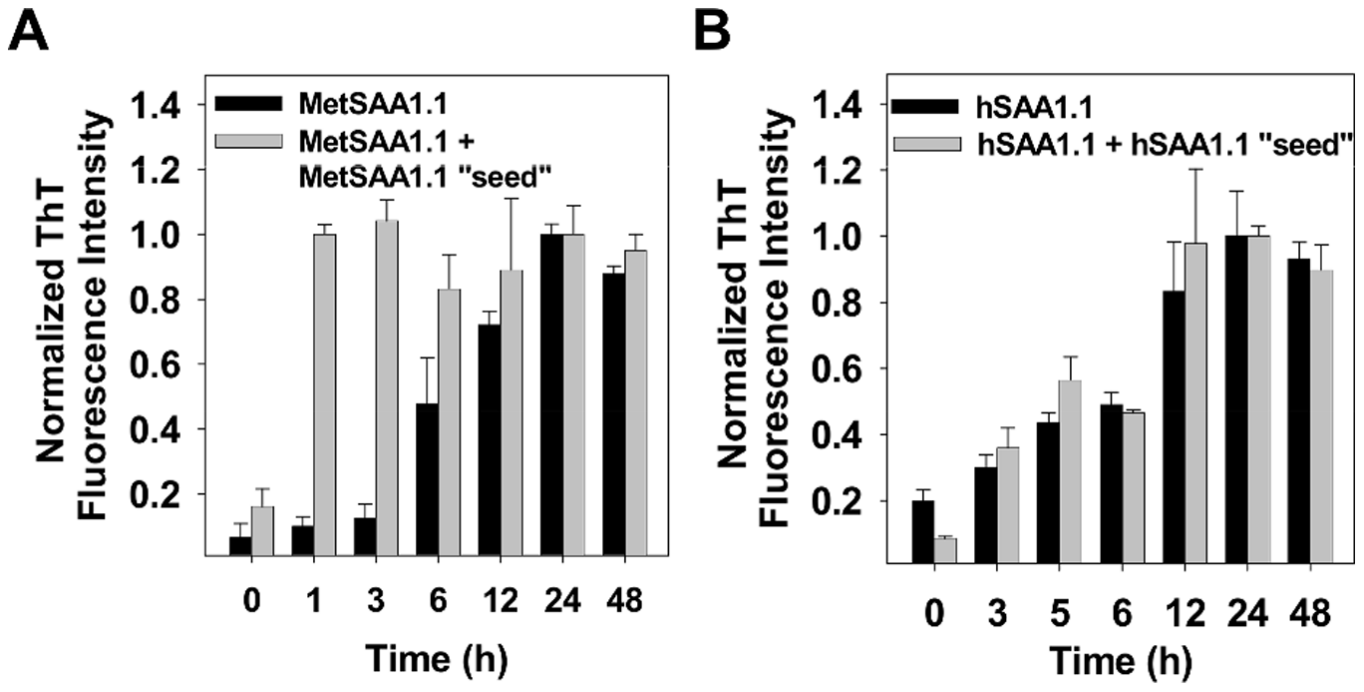
Characterization of “seeding” properties of MetSAA1.1 and hSAA1.1 by ThT fluorescence assay. (A) ThT fluorescence intensity profile for freshly refolded MetSAA1.1 only (black bars) and MetSAA1.1+ MetSAA1.1 “seed” (gray bars); (B) ThT fluorescence intensity profile for freshly refolded hSAA1.1 only (black bars) and hSAA1.1+ hSAA1.1 “seed” (gray bars). The concentration of protein was 20 µM. ThT fluorescence intensities were recorded by incubating the samples at 37°C.

After analyzing the fibrillation pathways of the two proteins, we then tested the ability of the aggregates formed by both the proteins to seed aggregation. Specifically, we attempted to “seed” the “native-like” oligomers of both MetSAA1.1 and hSAA1.1 with amyloid aggregates formed by the same protein following 72 h of incubation at 37°C. The solutions for these seeding experiments contained 18 µM freshly refolded MetSAA1.1 or hSAA1.1 and 2 µM MetSAA1.1 or hSAA1.1 aggregates obtained after 72 h incubation at 37°C. The final concentration of proteins in each of these mixtures was thus 20 µM (the same as that used for all previous studies) and the percentage of “seed” was effectively 10% of the total protein concentration. We monitored the aggregation kinetics of the “seeded” protein solutions using the ThT fluorescence assay and compared them with those for protein solutions containing 20 µM MetSAA1.1 or hSAA1.1 alone. As shown in Figure 4A (black bars) and also as observed previously (Fig. 2A), MetSAA1.1 aggregation was a gradual process with ThT fluorescence intensity saturating after ca. 24 h. “Seeding” freshly refolded MetSAA1.1 with MetSAA1.1 amyloid fibrils however significantly enhanced the rate of formation of cross-beta-rich aggregates with ThT fluorescence intensities saturating by ca. 1 h (Fig. 4A). On the other hand, we observed that hSAA1.1 “seed” did not have a significant impact on the aggregation kinetics of hSAA1.1 and ThT fluorescence intensities for both, hSAA1.1 alone (black bars) and hSAA1.1 plus hSAA1.1 “seed” (gray bars), saturated by ca. 24 h (Fig. 4B). Interestingly, “cross-seeding” freshly refolded hSAA1.1 with MetSAA1.1 amyloid fibrils marginally enhanced the rate of formation of cross-beta-rich aggregates with ThT fluorescence intensities saturating by ca. 6 h (Fig. S7 in File S1). Taken together these results show that while the late stage amyloid aggregates formed by MetSAA1.1 promote the conversion of MetSAA1.1 to cross-beta-rich aggregates, the late-stage aggregates formed by hSAA1.1 do not have a significant effect on hSAA1.1 aggregation.

## Discussion

We observed that refolding of MetSAA1.1 and hSAA1.1 primarily resulted in the formation of “native-like” oligomers (Fig. 1B) and the oligomerization behavior was concentration dependent (Fig. S2 in File S1). We found that the “native-like” oligomers formed by both the proteins were alpha-helix-rich, had similar tertiary structure, and were marginally stable (Fig. 1C–F). We had previously shown that the mouse isoforms of SAA also exist as marginally stable alpha helical oligomers [31], [32], [45]. These results obtained using human SAA in the current study and murine SAA isoforms in our past studies may reflect a ubiquitous property of SAA proteins to exist as alpha helix-rich and marginally stable oligomers upon refolding, a property that can be attributed to the high level of sequence conservation observed amongst SAA isoforms found in different vertebrates.

**Figure 5:**
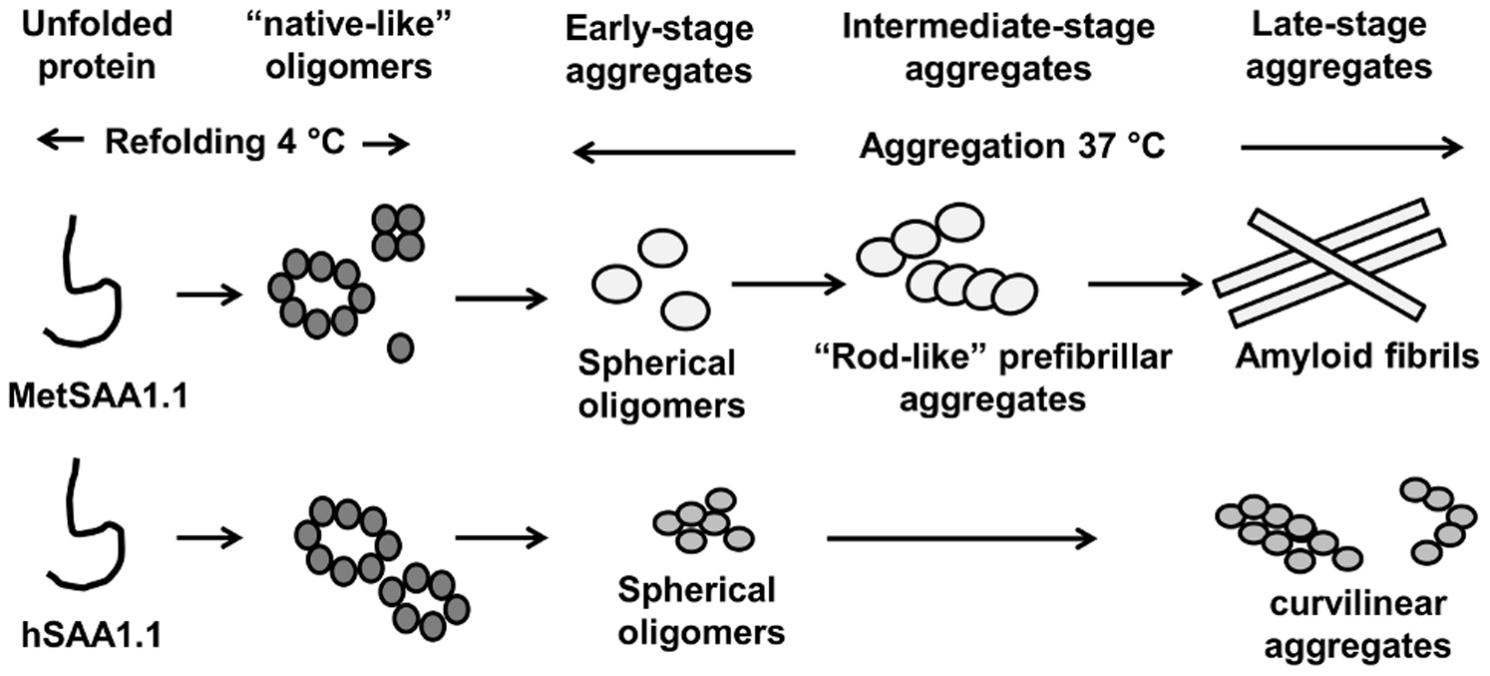
Cartoon representing the proposed pathway for oligomerization and fibrillation of MetSAA1.1 and hSAA1.1. Figures are not drawn to scale.

Our AFM studies revealed that hSAA1.1 did not form full-length amyloid fibrils upon incubation at 37°C even after ca. 200 h (Fig. 3D). The low amyloidogenicity of hSAA1.1 *in vitro* may seem surprising given its pathogenicity *in vivo*. This result is, however, consistent with our recent studies of murine SAA, where the pathogenic isoform SAA1.1 was found to be less amyloidogenic than the non-pathogenic isoform SAA2.2 [32]. Intriguingly, the addition of a single N-terminal methionine residue greatly enhanced the fibrillation propensity of hSAA1.1 and also modulated its fibrillation pathway. The proposed oligomerization and aggregation pathways for MetSAA1.1 and hSAA1.1 have been summarized in Figure 5. It would be interesting to probe the exact role of the methionine residue in modulating the amyloidogenicity of hSAA1.1 in future work. The ability of MetSAA1.1 to seed the aggregation of “native-like” oligomers of MetSAA1.1 suggests that the N-terminal region containing the methionine might be acting as a “template” for fibril formation. This result is consistent with previous studies that have indicated the importance of the N-terminus for fibril formation [46], and with the ability of N-terminal peptides to form fibrils [22]. However, caution must be exerted while correlating these studies to an *in vivo* model system especially since recombinantly expressed proteins lack the post-translational modifications typical of mammalian proteins.

Finally, analogous to the observed effect of a single N-terminal methionine *in vitro*, other factors may modulate the stability and fibrillation of SAA *in vivo*. In a previous study on a mouse isoform of SAA, we showed that Zn^2+^ binds to and stabilized the secondary, tertiary, and quaternary structure of mouse SAA2.2 while calcium destabilized SAA2.2 structure between 1 to 10 mM concentrations and induced its aggregation [47]. Heparan sulfate has also been shown to dissociate SAA from acute-phase HDL and promote its aggregation [48]. While the role of these co-factors and natural ligands remains to be completely elucidated, this study represents an important step in understanding the mechanism of hSAA1.1 oligomerization and fibrillation and may be useful for future studies of SAA-associated pathogenicity.

## Supporting Information

File S1 Figure S1, Monitoring TEV-assisted proteolysis of (His)_6_-TEV-hSAA1.1 to hSAA1.1 by SDS-PAGE: (lane 1), TEV protease; (lane 2), (His)_6_-TEV-hSAA1.1 obtained post-IMAC and SEC; (lane 3–8), TEV cleavage of (His)_6_-TEV-hSAA1.1 to generate hSAA1.1 monitored as a function of time; (lane 9) hSAA1.1 post-SEC purification. Figure S2, Influence of concentration on the oligomerization behavior of MetSAA1.1 and hSAA1.1: (A and B) SEC elution profiles of MetSAA1.1 and hSAA1.1 respectively refolded at 75 µM (black solid line), 20 µM (blue solid line), and 8.5 µM (red solid line) concentrations; (C and D) Far-UV CD-based thermal denaturation profiles of MetSAA1.1 and hSAA1.1 respectively at 20 µM (blue solid line) and 8.5 µM (green solid line) concentrations; (E and F) Tryptophan fluorescence emission spectra of MetSAA1.1 and hSAA1.1 respectively at 20 µM (blue solid line) and 8.5 µM (green solid line) concentrations. Figure S3, Characterization of residual structure of MetSAA1.1 and hSAA1.1 in urea solution: SEC elution profiles of (A) MetSAA1.1 (blue solid line) and (B) hSAA1.1 (red solid line) in 8 M urea solution; (C) Far-UV CD of MetSAA1.1 in 0 M (black solid line), 1 M (blue solid line), and 2 M (red solid line) urea solutions; (D) Far-UV CD of hSAA1.1 in 0 M (black solid line), 1 M (blue solid line), and 2 M (red solid line) urea solutions. Concentration of proteins used for all the assays was 20 µM. Assays were performed at 4°C. Figure S4, “Cross-purification” studies to analyze the oligomerization behavior of MetSAA1.1 and hSAA1.1: SEC elution profiles of (A) “regular” MetSAA1.1 (blue solid line) and “cross-purified” MetSAA1.1 (red solid line); (B) “regular” hSAA1.1 (blue solid line) and “cross-purified” hSAA1.1 (red solid line). Concentration of proteins used was 20 µM. Assays were performed at 4°C. Figure S5, Biophysical characterization of “native-like” oligomers formed by MetSAA1.1 and hSAA1.1: AFM analysis of (A) MetSAA1.1 “native-like” oligomers; (B) hSAA1.1 “native-like” oligomers. All scale bars for AFM images represent 1 µm. Figure S6, Biophysical characterization of aggregates formed by MetSAA1.1 and hSAA1.1: AFM analysis of (A) MetSAA1.1, 6 h, 37°C; (B) MetSAA1.1, 24 h, 37°C; (C) hSAA1.1, 12 h, 37°C; (D) hSAA1.1, 48 h, 37°C. All scale bars for AFM images represent 1 µm. Figure S7, Characterization of “cross-seeding” properties of MetSAA1.1 by ThT fluorescence assay: ThT fluorescence intensity profile for freshly refolded hSAA1.1 only (black bars) and hSAA1.1+ MetSAA1.1 “seed” (gray bars. The concentration of protein was 20 µM. ThT fluorescence intensities were recorded by incubating the samples at 37°C.
(DOC)
