# Supplementary material for: Characterization of the Oligomerization and Aggregation of Human Serum Amyloid A
Source: PLoS One. 2013 Jun 4;8(6):e64974. doi: 10.1371/journal.pone.0064974 (PMC3672174; doi:10.1371/journal.pone.0064974)
Supplement: File S1 — Figure S1, Monitoring TEV-assisted proteolysis of (His)6-TEV-hSAA1.1 to hSAA1.1 by SDS-PAGE: (lane 1), TEV protease; (lane 2), (His)6-TEV-hSAA1.1 obtained post-IMAC and SEC; (lane 3–8), TEV cleavage of (His)6-TEV-hSAA1.1 to generate hSAA1.1 monitored as a function of time; (lane 9) hSAA1.1 post-SEC purification. Figure S2, Influence of concentration on the oligomerization behavior of MetSAA1.1 and hSAA1.1: (A and B) SEC elution profiles of MetSAA1.1 and hSAA1.1 respectively refolded at 75 µM (black solid line), 20 µM (blue solid line), and 8.5 µM (red solid line) concentrations; (C and D) Far-UV CD-based thermal denaturation profiles of MetSAA1.1 and hSAA1.1 respectively at 20 µM (blue solid line) and 8.5 µM (green solid line) concentrations; (E and F) Tryptophan fluorescence emission spectra of MetSAA1.1 and hSAA1.1 respectively at 20 µM (blue solid line) and 8.5 µM (green solid line) concentrations. Figure S3, Characterization of residual structure of MetSAA1.1 and hSAA1.1 in urea solution: SEC elution profiles of (A) MetSAA1.1 (blue solid line) and (B) hSAA1.1 (red solid line) in 8 M urea solution; (C) Far-UV CD of MetSAA1.1 in 0 M (black solid line), 1 M (blue solid line), and 2 M (red solid line) urea solutions; (D) Far-UV CD of hSAA1.1 in 0 M (black solid line), 1 M (blue solid line), and 2 M (red solid line) urea solutions. Concentration of proteins used for all the assays was 20 µM. Assays were performed at 4°C. Figure S4, “Cross-purification” studies to analyze the oligomerization behavior of MetSAA1.1 and hSAA1.1: SEC elution profiles of (A) “regular” MetSAA1.1 (blue solid line) and “cross-purified” MetSAA1.1 (red solid line); (B) “regular” hSAA1.1 (blue solid line) and “cross-purified” hSAA1.1 (red solid line). Concentration of proteins used was 20 µM. Assays were performed at 4°C. Figure S5, Biophysical characterization of “native-like” oligomers formed by MetSAA1.1 and hSAA1.1: AFM analysis of (A) MetSAA1.1 “native-like” oligomers; (B) hSAA1.1 “native-l [file pone.0064974.s001.doc]

Characterization of the Oligomerization and Aggregation of Human Serum Amyloid A

Sanket Patke^1,2^, Saipraveen Srinivasan^2^, Ronak Maheshwari^2^, Sunit K. Srivastava^2^, J. Javier Aguilera^2,3^, Wilfredo Colón^2,3^*, and Ravi S. Kane^1,2^*

^1^Department of Chemical and Biological Engineering, ^2^Center for Biotechnology and Interdisciplinary Studies, and ^3^Department of Chemistry and Chemical Biology, Rensselaer Polytechnic Institute, Troy, New York 12180

* To whom correspondence should be addressed: Wilfredo Colón, 110 8^th^ St., Troy, NY, USA / Ravi S. Kane, 110 8^th^ St., Troy, NY, USA, 12180. Email: colonw@rpi.edu; kaner@rpi.edu. Tel:  [(518) 276-2515](tel:(518) 276-2515); [(518) 276-2536](tel:(518) 276-2536).

**MetSAA1.1 PROTEIN SEQUENCE:**

M R S F F S F L G E A F D G A R D M W R A Y S D M R E A N Y I G S D K Y F H A R G N Y D A A K R G P G G V W A A E A I S D A R E N I Q R F F G H G A E D S L A D Q A A N E W G R S G K D P N H F R P A G L P E K Y

Number of Amino acids: 105

Molecular Weight: 11.815 kDa

Extinction Co-efficient^#^: 23950 cm^-1^M^-1^

**hSAA1.1 PROTEIN SEQUENCE:**

R S F F S F L G E A F D G A R D M W R A Y S D M R E A N Y I G S D K Y F H A R G N Y D A A K R G P G G V W A A E A I S D A R E N I Q R F F G H G A E D S L A D Q A A N E W G R S G K D P N H F R P A G L P E K Y

Number of Amino acids: 104

Molecular Weight: 11.684 kDa

Extinction Co-efficient^#^: 23950 cm^-1^M^-1^

# Values of Extinction Coefficient for individual proteins were obtained using the ExPASy ProtParam web server [1] with known protein sequence

1. Wilkins MR, Gasteiger E, Bairoch A, Sanchez JC, Williams KL, et al. (1999) Protein identification and analysis tools in the ExPASy server. Methods Mol Biol 112: 531-552.

**UNITPROTKB OF hSAA1.1**

locus SAA1_HUMAN, accession [P0DJI8](http://www.uniprot.org/uniprot/P0DJI8)

**CLEAVAGE SITE OF TEV IN (HIS)_6_-TEV-hSAA1.1 PROTEIN SEQUENCE**

MAHHHHHHSAGENLYF**Q^R**SFFSFLGEAFDGARDMWRAYSDMREANYIGSDKYFHARGNYDAAKRGPGGVWAAEAISDARENIQRFFGHGAEDSLADQAANEWGRSGKDPNHFRPAGLPEKY

“**^**” between residues Q (residue number 17) and R (residue number 18) represents the site where TEV cleaves (HIS)_6_-TEV-hSAA1.1


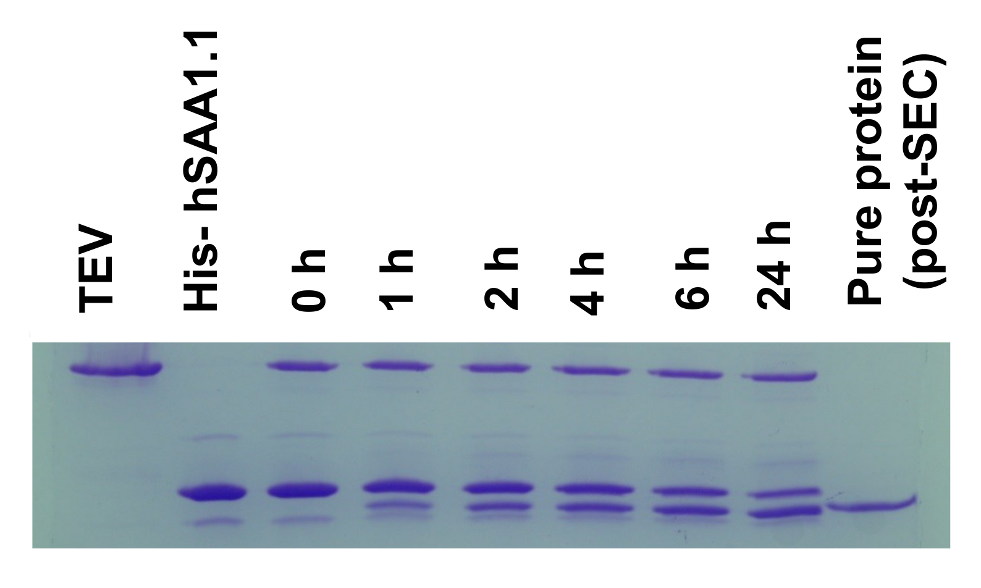


Figure S1. **Monitoring TEV-assisted proteolysis of (His)_6_-TEV-hSAA1.1 to hSAA1.1 by SDS-PAGE**: (lane 1), TEV protease; (lane 2), (His)_6_-TEV-hSAA1.1 obtained post-IMAC and SEC; (lane 3 – 8), TEV cleavage of (His)_6_-TEV-hSAA1.1 to generate hSAA1.1 monitored as a function of time; (lane 9) hSAA1.1 post-SEC purification.


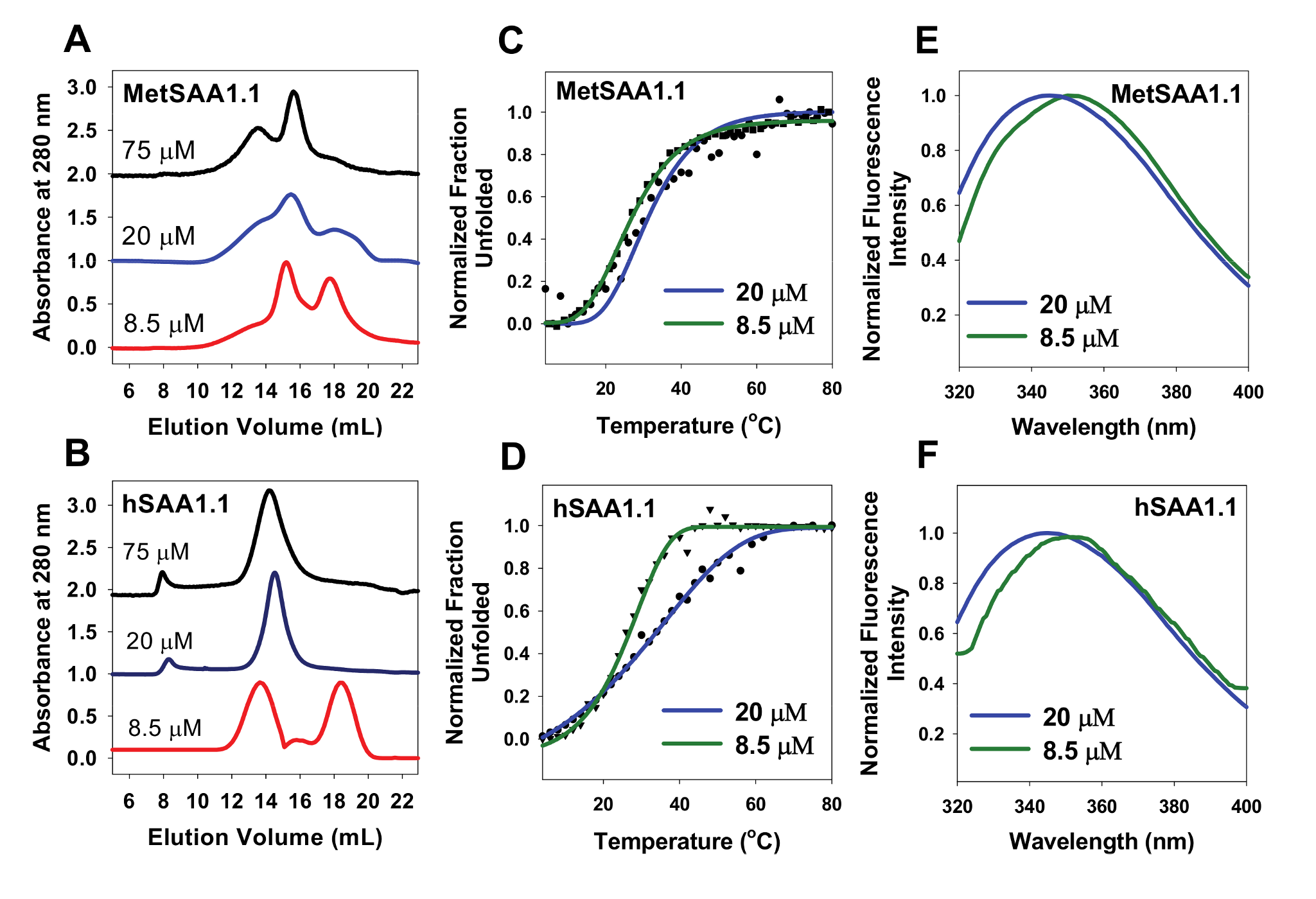


Figure S2. **Influence of concentration on the oligomerization behavior of MetSAA1.1 and hSAA1.1**: (A and B) SEC elution profiles of MetSAA1.1 and hSAA1.1 respectively refolded at 75 µM (black solid line), 20 µM (blue solid line), and 8.5 µM (red solid line) concentrations; (C and D) Far-UV CD-based thermal denaturation profiles of MetSAA1.1 and hSAA1.1 respectively at 20 µM (blue solid line) and 8.5 µM (green solid line) concentrations; (E and F) Tryptophan fluorescence emission spectra of MetSAA1.1 and hSAA1.1 respectively at 20 µM (blue solid line) and 8.5 µM (green solid line) concentrations.


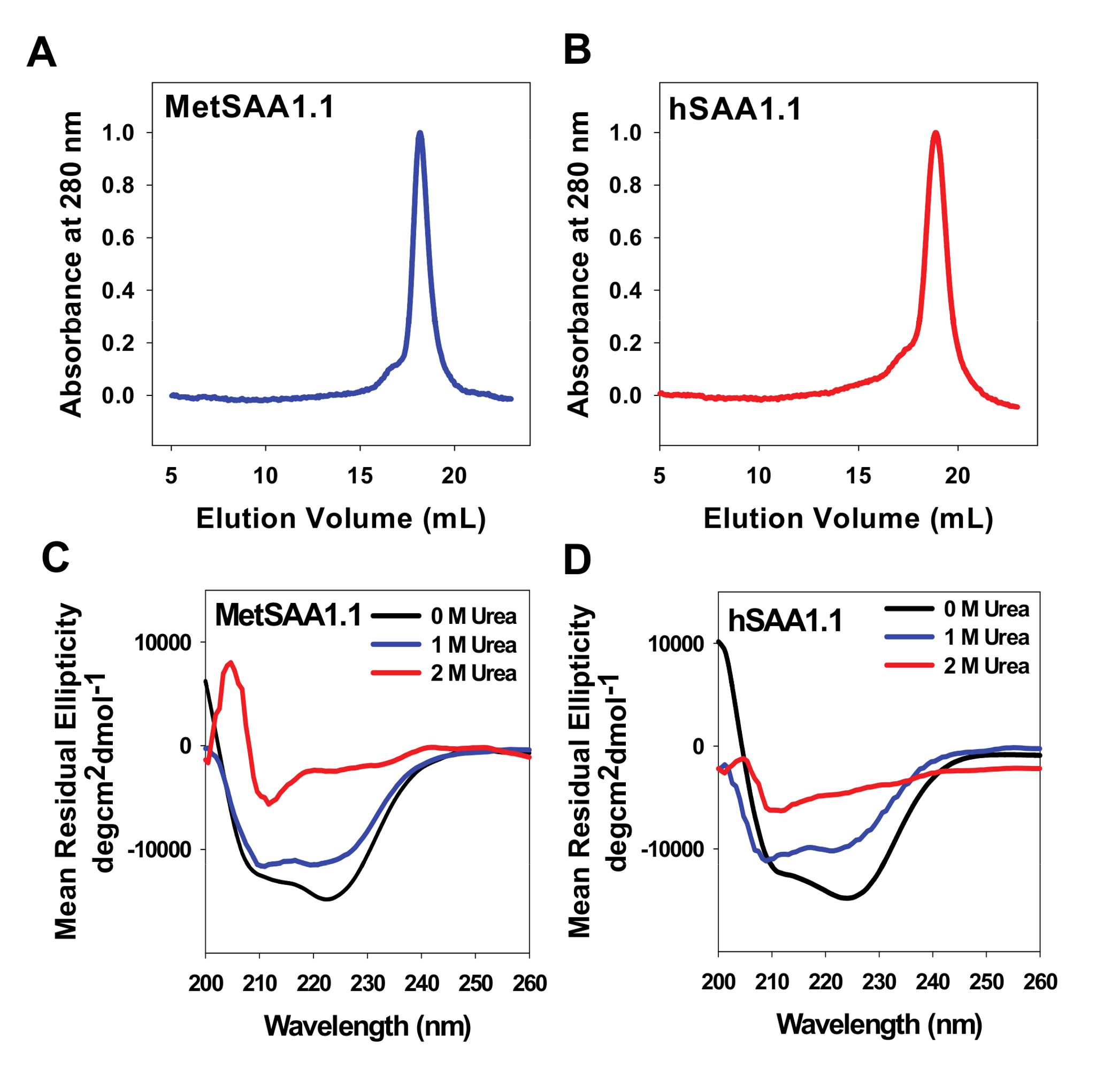


Figure S3. **Characterization of residual structure of MetSAA1.1 and hSAA1.1 in urea solution**: SEC elution profiles of (A) MetSAA1.1 (blue solid line) and (B) hSAA1.1 (red solid line) in 8 M urea solution; (C) Far-UV CD of MetSAA1.1 in 0 M (black solid line), 1 M (blue solid line), and 2 M (red solid line) urea solutions; (D) Far-UV CD of hSAA1.1 in 0 M (black solid line), 1 M (blue solid line), and 2 M (red solid line) urea solutions. Concentration of proteins used for all the assays was 20 µM. Assays were performed at 4 ºC.


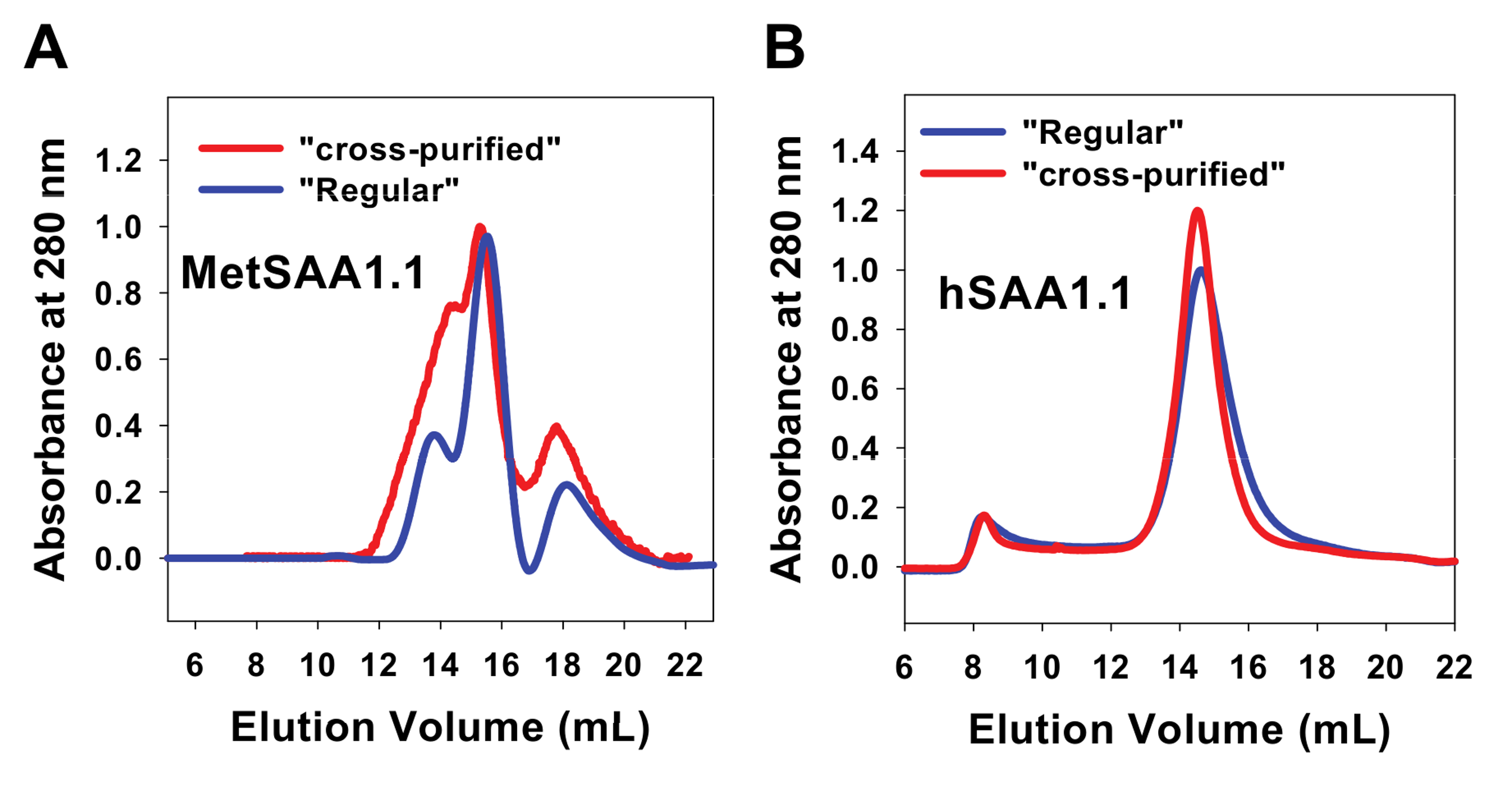


Figure S4. **“Cross-purification” studies to analyze the oligomerization behavior of MetSAA1.1 and hSAA1.1**: SEC elution profiles of (A) “regular” MetSAA1.1 (blue solid line) and “cross-purified” MetSAA1.1 (red solid line); (B) “regular” hSAA1.1 (blue solid line) and “cross-purified” hSAA1.1 (red solid line). Concentration of proteins used was 20 µM. Assays were performed at 4 ºC.


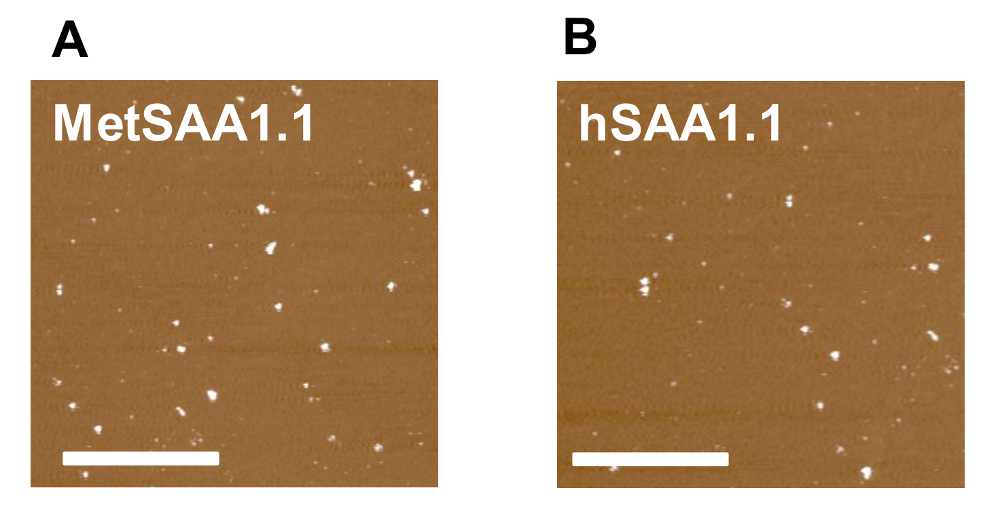


**MetSAA1.1**

**hSAA1.1**

Figure S5. **Biophysical characterization of “native-like” oligomers formed by MetSAA1.1 and hSAA1.1**: AFM analysis of (A) MetSAA1.1 “native-like” oligomers; (B) hSAA1.1 “native-like” oligomers. All scale bars for AFM images represent 1 μm.


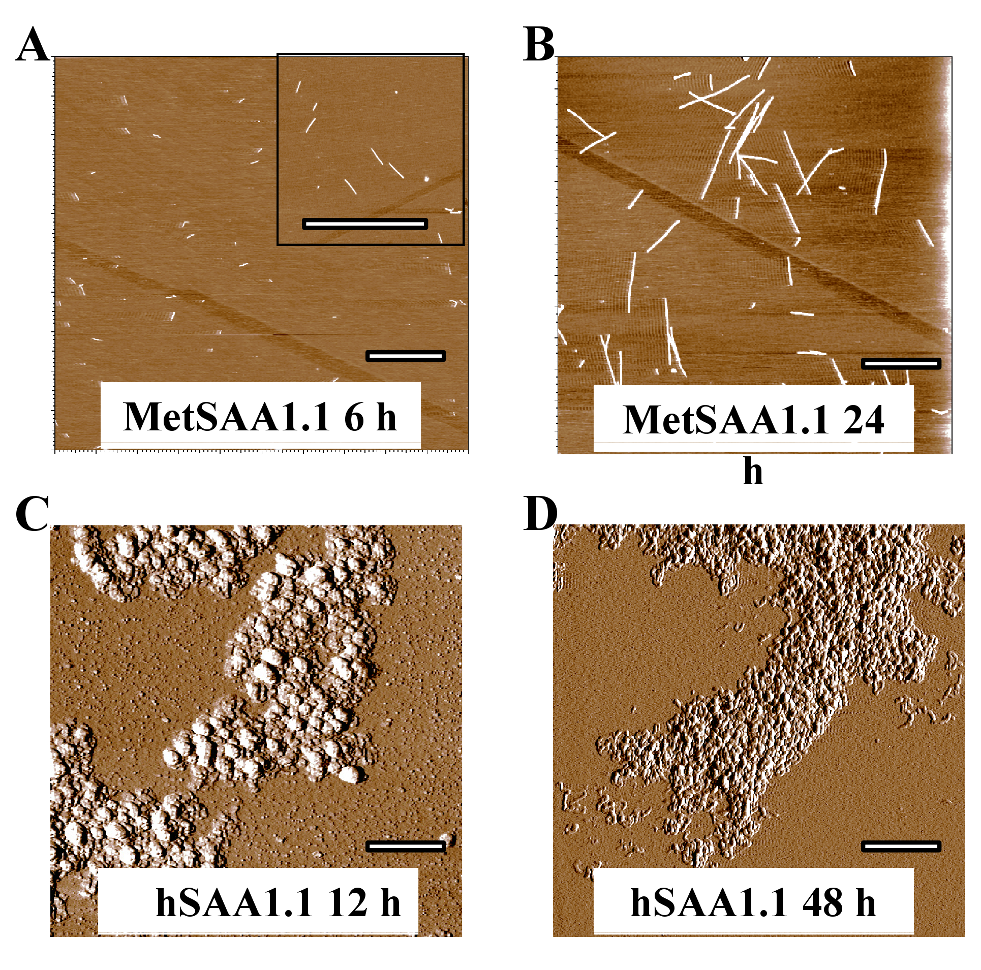


Figure S6. **Biophysical characterization of aggregates formed by MetSAA1.1 and hSAA1.1**: AFM analysis of (A) MetSAA1.1, 6 h, 37 °C; (B) MetSAA1.1, 24 h, 37 °C; (C) hSAA1.1, 12h, 37 °C; (D) hSAA1.1, 48 h, 37 °C. All scale bars for AFM images represent 1 μm.


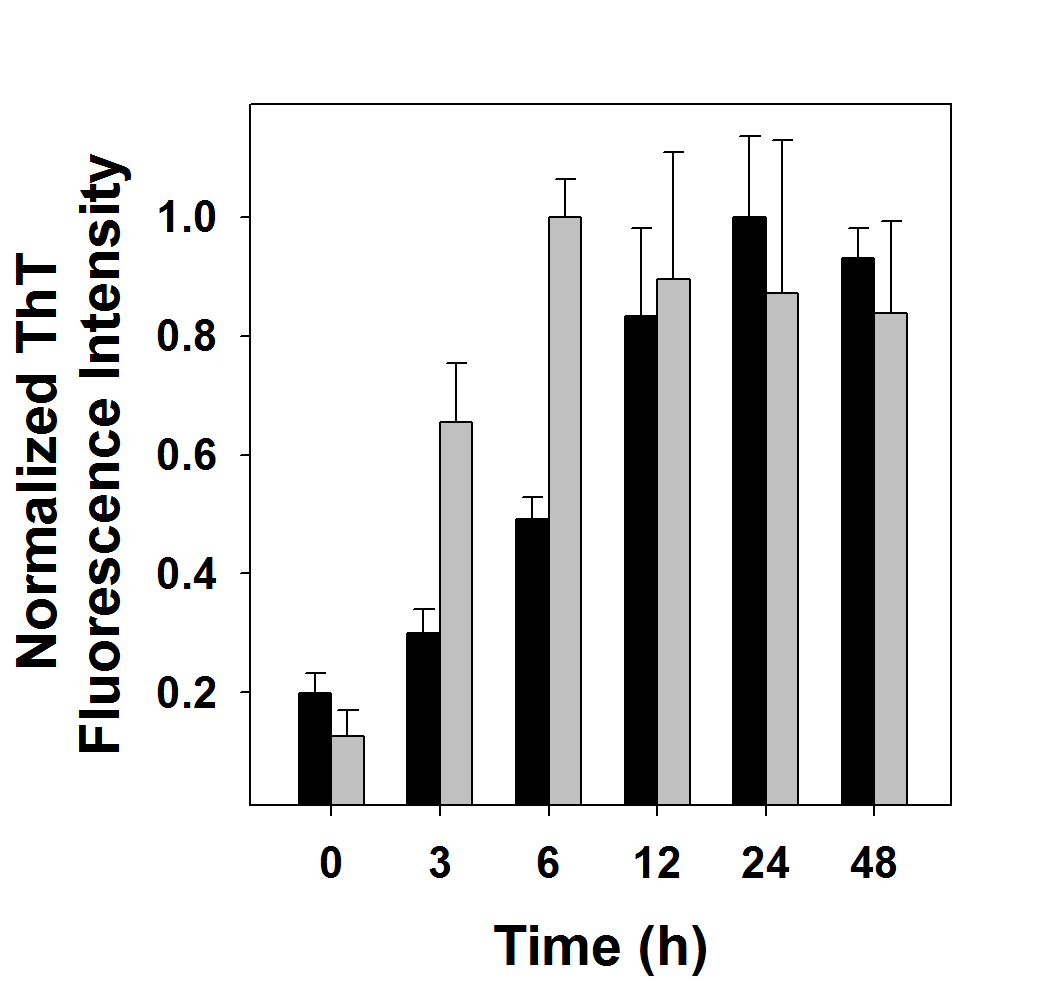


Figure S7. **Characterization of “cross-seeding” properties of MetSAA1.1 by ThT fluorescence assay**: ThT fluorescence intensity profile for freshly refolded hSAA1.1 only (black bars) and hSAA1.1 + MetSAA1.1 “seed” (gray bars. The concentration of protein was 20 μM. ThT fluorescence intensities were recorded by incubating the samples at 37 °C.
